# Diagnosis of Autoimmune Blistering Diseases

**DOI:** 10.3389/fmed.2018.00296

**Published:** 2018-11-02

**Authors:** Mareike Witte, Detlef Zillikens, Enno Schmidt

**Affiliations:** Department of Dermatology, University of Lübeck, Lübeck, Germany

**Keywords:** autoantibody, biochip, immunofluorescence, ELISA, pemphigus, pemphigoid, epidermolysis bullosa acquisita, dermatitis herpetiformis

## Abstract

Autoimmune skin blistering diseases (AIBD) are characterized by autoantibodies that are directed against structural proteins in the skin and adjacent mucous membranes. Some clinical signs are typical for a specific AIBD, however, correct diagnosis requires the detection of tissue-bound or circulating autoantibodies. The gold standard for diagnosis of AIBD is the detection of autoantibodies or complement component 3 by direct immunofluorescence (DIF) microscopy of a perilesional biopsy. Circulating antibodies can be detected via indirect immunofluorescence (IIF) microscopy of different tissue substrates including human skin, monkey esophagus, and more recently, recombinant forms of the different target antigens. Latter are also employed in various commercial ELISA systems and by immunoblotting in in-house assays available in specialized laboratories. ELISA systems are also particularly valuable for monitoring of the disease activity during the disease course which can be helpful for treatment decisions. Exact diagnosis is essential for both treatment and prognosis, since some AIBD are associated with malign tumors such as paraneoplastic pemphigus and anti-laminin 332 mucous membrane pemphigoid. This review presents clinical and immunopathological features of AIBD for the state-of the art diagnosis of these disorders.

## Introduction

Autoimmune skin blistering diseases (AIBD) are a diverse group of dermatoses that are characterized by autoantibodies binding to antigens in the skin and mucous membranes. They can be subdivided into pemphigoid diseases (PD), with subepidermal split formation and autoantibody binding to structural components of the dermal-epidermal junction (DEJ), and pemphigus, with autoantibodies directed against desmosomal proteins that connect neighboring keratinocytes (1, 2). A special type of AIBD is dermatitis herpetiformis, with autoantibodies directed against the tissue and epidermal transglutaminase. In this review, we will provide a comprehensive overview about the clinical features and current diagnosis of AIBD extending and updating previous work (3, 4).

## Epidemiology

Bullous pemphigoid (BP) is the most frequent AIBD in Central Europe. Its incidence reaches around 20/million/year. BP is followed by mucous membrane pemphigoid (MMP) and pemphigoid gestationis, with incidences of 2/million/year, respectively (5–8). Higher incidences of BP have been reported in Great Britain (9). In contrast to other autoimmune diseases, the incidence of BP is increasing with age. Regarding this matter, its annual incidence in people older than 80 years reaches 150–180/million/year (5, 6). Like other autoimmune diseases, the incidence of BP is constantly increasing and has nearly doubled in the last decade (7–11). This is partly due to the rising life expectancy of the general population, increasing awareness, and enhanced diagnostic tests. Further, the close association between BP and neurological diseases [reviewed in (12)], whose incidences are also rising, may contribute to the increased occurrence of BP. This rise in BP incidence is reflected by hospitalization numbers of BP patients that increased by 26% for a primary diagnosis and by 62% for a secondary diagnosis to 3,260/million inpatients between 2002 and 2012 in the USA (13).

In pemphigus, the incidence depends on the geographical region. In Central Europe and the United States, its incidence is estimated between 1 and 7 new patients/million/year (9, 14). Generally, PV is more common than pemphigus foliaceus (PF), with ratios ranging from 4:1 to 9:1 (15). In Tunisia and Brazil, endemic forms of PF with much higher incidences are present (16, 17).

The prevalence of AIBD in Germany have recently been calculated based on the ICD-coding-based dataset of the country's largest health insurance. The study revealed about 40,000 AIBD patients including 21,000 patients with BP, 7,700 with PV, and around 2,000 with MMP (18).

## Historical background

The term pemphigus was first used by Hippocrates in 460–370 B.C. (19). However, the differentiation between pemphigus and BP was first made by Walter Lever in 1953 based on lesional histopathology (20). In 1964 and 1967, detection of autoantibodies in serum and skin were reported for pemphigus and BP (21, 22), providing milestones for the diagnosis of AIBD. Diagnosis of the different AIBD entities became subsequently possible by the molecular identification of target antigens (23). In parallel it became clear that the autoantibodies used for the diagnosis of AIBDs may be directly pathogenic (24–29), reviewed in (1, 30–34).

## Direct immunofluorescence microscopy

The diagnosis of AIBDs is based on the combination of the clinical presentation and detection of tissue-bound and/or circulating autoantibodies. Tissue-bound autoantibodies can be detected via direct immunofluorescence (DIF) microscopy, which is the diagnostic gold standard for AIBD. For DIF microscopy, cryosections of perilesional biopsies are required and need to be snap frozen and stored at −20°C or conserved in isotonic NaCl or modified Michels medium until processed (35, 36).

**Figure 1:**
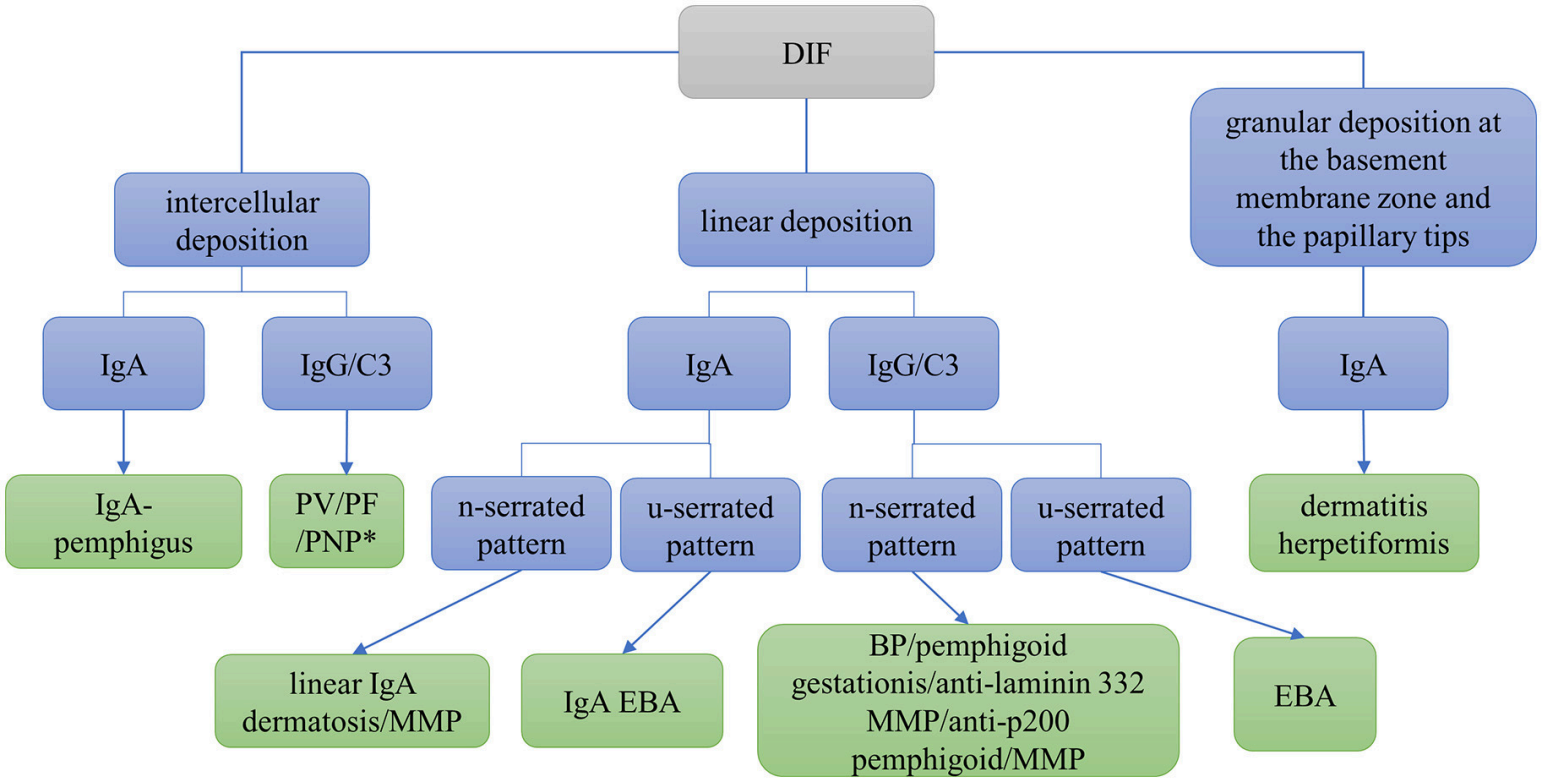
Differential diagnosis of autoimmune skin blistering diseases based on direct immunofluorescence (DIF) microscopy of a perilesional biopsy. BP, bullous pemphigoid; EBA, epidermolysis bullosa acquisita; MMP, mucous membrane pemphigoid; PF, pemphigus foliaceus; PNP, paraneoplastic pemphigus; PV, pemphigus vulgaris; *may be combined with linear deposition.

**Figure 2:**
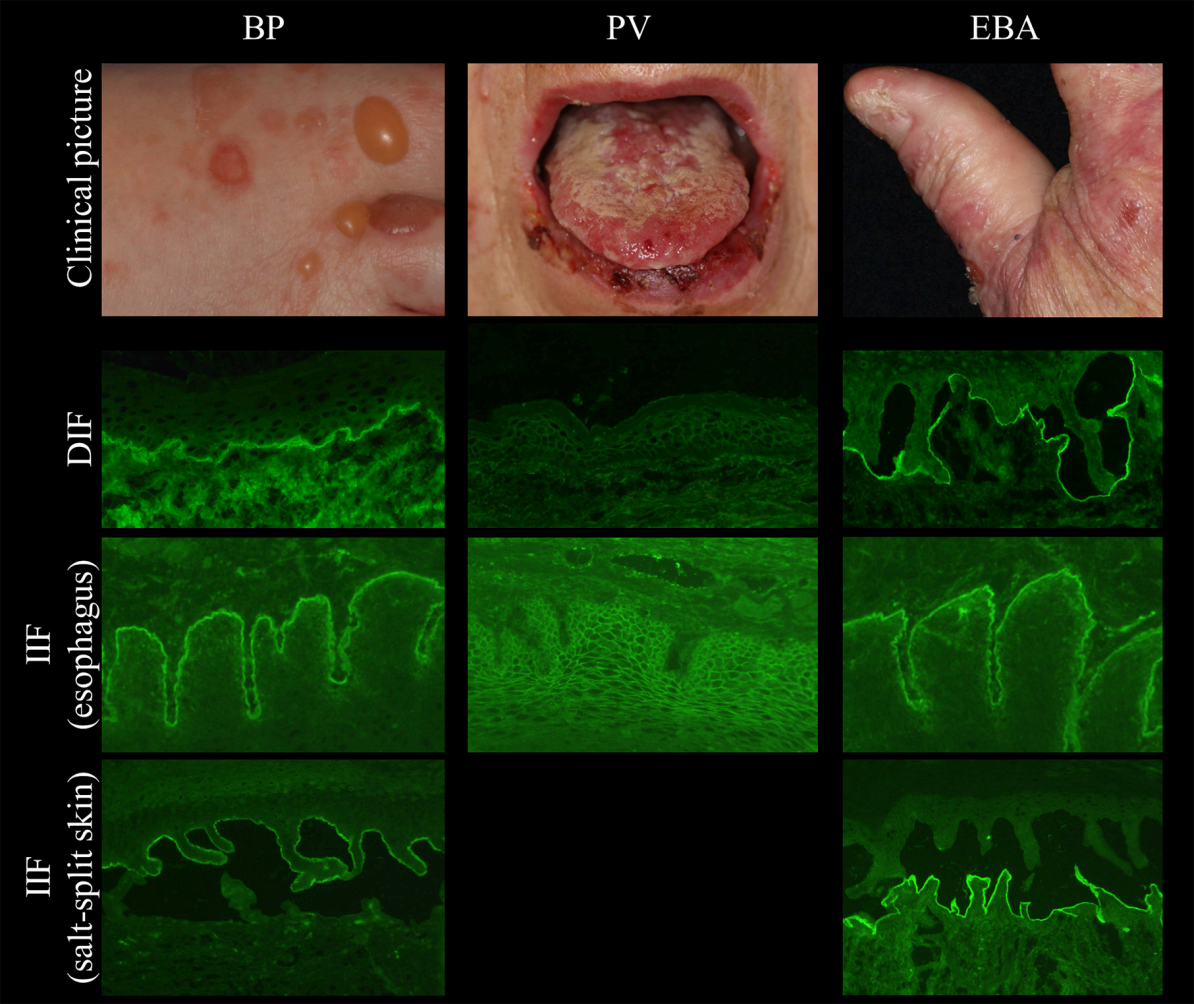
Clinical and immunopathological characteristics in bullous pemphigoid (BP, **left**), pemphigus vulgaris (PV, **middle**), and epidermolysis bullosa acquisita (EBA, **right**).

**Figure 3:**
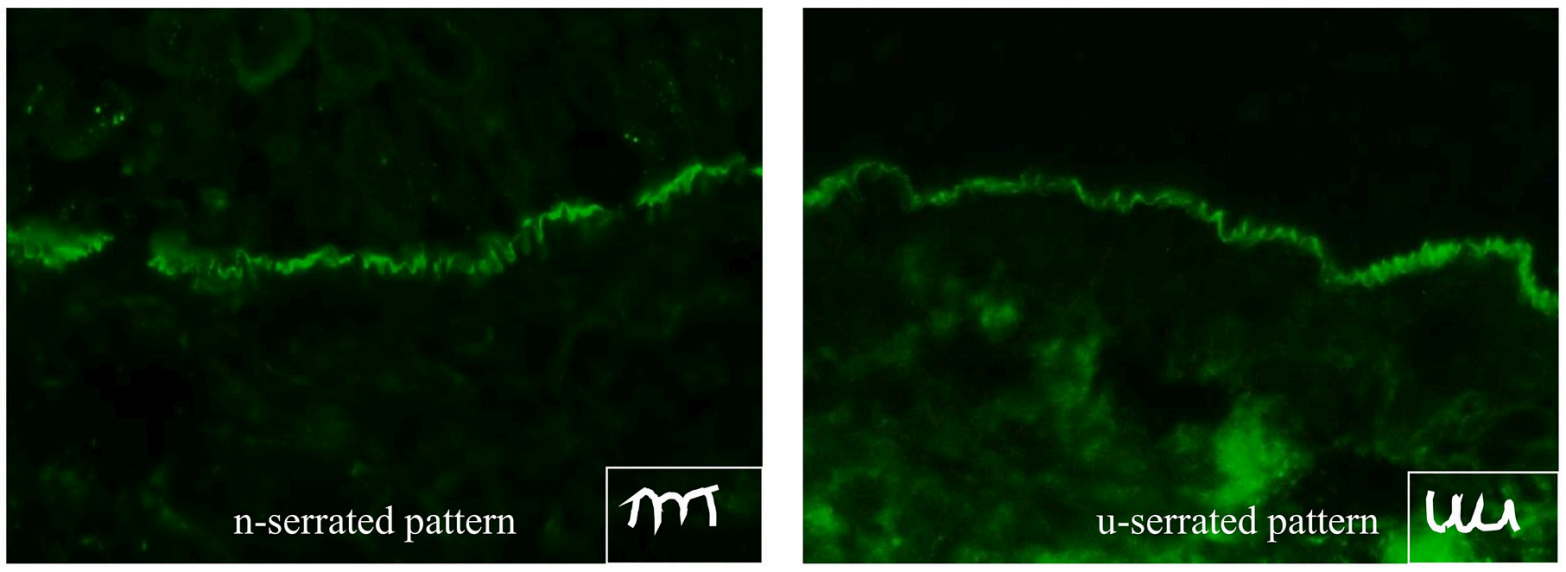
n-serrated **(left)** and u-serrated pattern **(right)** of basement membrane zone staining in pemphigoid diseases detected by direct immunofluorescence microscopy. While an u-serrated pattern is exclusively seen in epidermolysis bullosa acquisita, an n-serrated pattern can be detected in all other pemphigoid diseases.

DIF microscopy only provides limited information about the target antigen(s), however the diagnosis can be narrowed down according to the immunoglobulin subclass and binding pattern. In pemphigus, DIF microscopy reveals intercellular binding of IgG and/or C3 within the epidermis and/or epithelium. In pemphigoid diseases, a linear deposition of IgG and/or C3 at the DEJ can be observed (Figures 1, 2). Linear staining at the DEJ can further be differentiated into n-serrated and u-serrated patterns. In an n-serration pattern, arches are closed at the top (Figure 3 left) and in a u-serrated staining pattern, arches are closed at the bottom appearing like “growing grass” (Figure 3 right). While u-serration is unique for antibody binding to type VII collagen and can been seen in epidermolysis bullosa acquisita (EBA), n-serration is found in all other pemphigoid diseases (37–39). Serration pattern analysis can be performed in any routine immunofluorescence (IF) laboratory and is best performed in 6 μm sections and 400- or 600-fold magnification without oil (38, 39). IF pictures for training of serration pattern analysis are freely available (www.nversusu.umcg.nl).

In dermatitis herpetiformis, DIF microscopy reveals granular deposition of IgA at the dermal papillae and along the DEJ. An automated staining for DIF microscopy sections has recently been developed and revealed more intensive IF staining and reduced background compared to the manual procedure due to continuous movement and overhead incubation (40). A flowchart navigating through differential diagnoses using DIF microscopy is shown in Figure 1. For further differentiation of the target antigen(s) serological analyses is required.

## Indirect immunofluorescence microscopy using tissue substrates

Several tissues can be employed by indirect IF (IIF) microscopy to screen for serum autoantibodies in AIBD including monkey, rabbit, guinea pig, and human esophagus (for pemphigus and pemphigoid diseases), monkey and rat bladder (for paraneoplastic pemphigus), and amnion epithelium (for BP and PV). In one study, monkey esophagus was the most sensitive substrate for pemphigus; another study showed that monkey esophagus is more sensitive for PV and human esophagus is more sensitive for PF (41–44). The most frequently used substrates are monkey esophagus and human split skin. On monkey esophagus, autoantibodies in pemphigus reveal intercellular labeling of the epithelium and linear staining of the DEJ in pemphigoid diseases (Figure 2). Sensitivities of 90% and 73.2% have been reported for pemphigus and BP, respectively (41, 43). In dermatitis herpetiformis, IgA binds to the endomysium. The tissue substrate with the highest sensitivity for autoantibodies in pemphigoid diseases is 1M NaCl split human skin. Here, antibodies bind either to the epidermal (“roof”) or dermal (“floor”) side of the artificial blister (Figure 2, left and right panel, respectively). “Floor”-binding antibodies can be detected in EBA, anti-p200/laminin γ1 pemphigoid, and anti-laminin 332 MMP. “Roof”-binding antibodies target BP180 and BP230 and are observed in BP, linear IgA-disease, pemphigoid gestationis, and anti-BP180-type mucous membrane pemphigoid. Sensitivities for BP range between 73 and 84% (41, 45).

**Figure 4:**
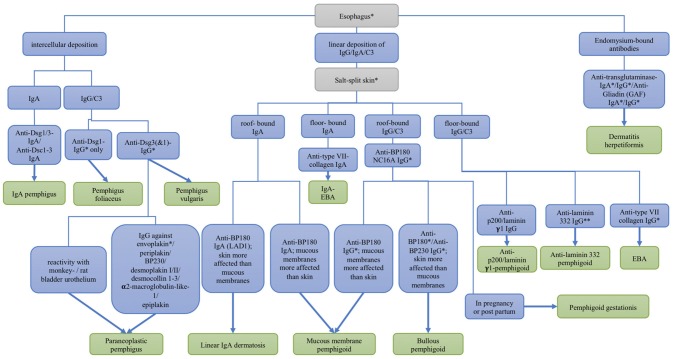
Differential diagnosis of autoimmune skin blistering diseases based on the serological detection of autoantibodies. Dsc, desmocollin; Dsg, desmoglein; EBA, epidermolysis bullosa acquisita; * commercial assays are available; ** available by the end of 2018.

The most sensitive substrates for the detection of anti-plakin reactivity are monkey and rat bladder epithelium. In pemphigoid gestationis, the complement fixation test detects complement-fixing IgG on human salt-split skin. For definite diagnosis of most AIBD refined analysis of serum autoantibodies can be performed, employing recombinant or cell-derived antigens. A flowchart depicting the serological diagnosis of autoimmune blistering diseases is shown in Figure 4.

## Target antigen-specific analysis of serum autoantibodies

For the identification of the target antigen, three main systems have been described: (i) Enzyme-linked immunosorbent assay (ELISA), (ii) IIF microscopy, and (iii) immunoblot/immunoprecipitation.

- ELISA systems allow the identification and quantification of autoantibodies against specific autoantigens. They are applied for both diagnosis and monitoring of the activity of the disease during the disease process (46). For pemphigoid diseases, commercial ELISA systems include BP180 NC16A, BP230, and type VII collagen, which employ recombinant protein, respectively (MBL, Euroimmun) (47–52). The sensitivity of the BP180 NC16A ELISA ranges between 84 and 89% in BP (47, 49, 53) and between 96 and 97% in pemphigoid gestations (54, 55). The sensitivity in BP can be increased by the additional use of the BP230 ELISA by about 5% (52, 56, 57). For pemphigus, ELISA systems employ the ectodomains of Dsg1 and Dsg3 recombinantly expressed in HEK293 cells (Euroimmun) or baculovirus (MBL, Nagoya, Japan) (46, 58, 59). For paraneoplastic pemphigus, an ELISA system for autoantibodies against envoplakin has been developed (Euroimmun) (60). For dermatitis herpetiformis, ELISA systems for the detection of coeliac-specific gliadin IgG and IgA autoantibodies as well as anti-transglutaminase 2 and 3-antibodies are available (61). ELISA systems that are less standardized and only available in specialized laboratories include desmocollin (62, 63), laminin γ1 (64, 65), the ectodomain of BP180 (66), full-length BP180 (67), laminin 332 (68, 69) and BP180 NC16A-IgE-ELISA (70–72) as well as other forms of BP180 (73, 74).

**Figure 5:**
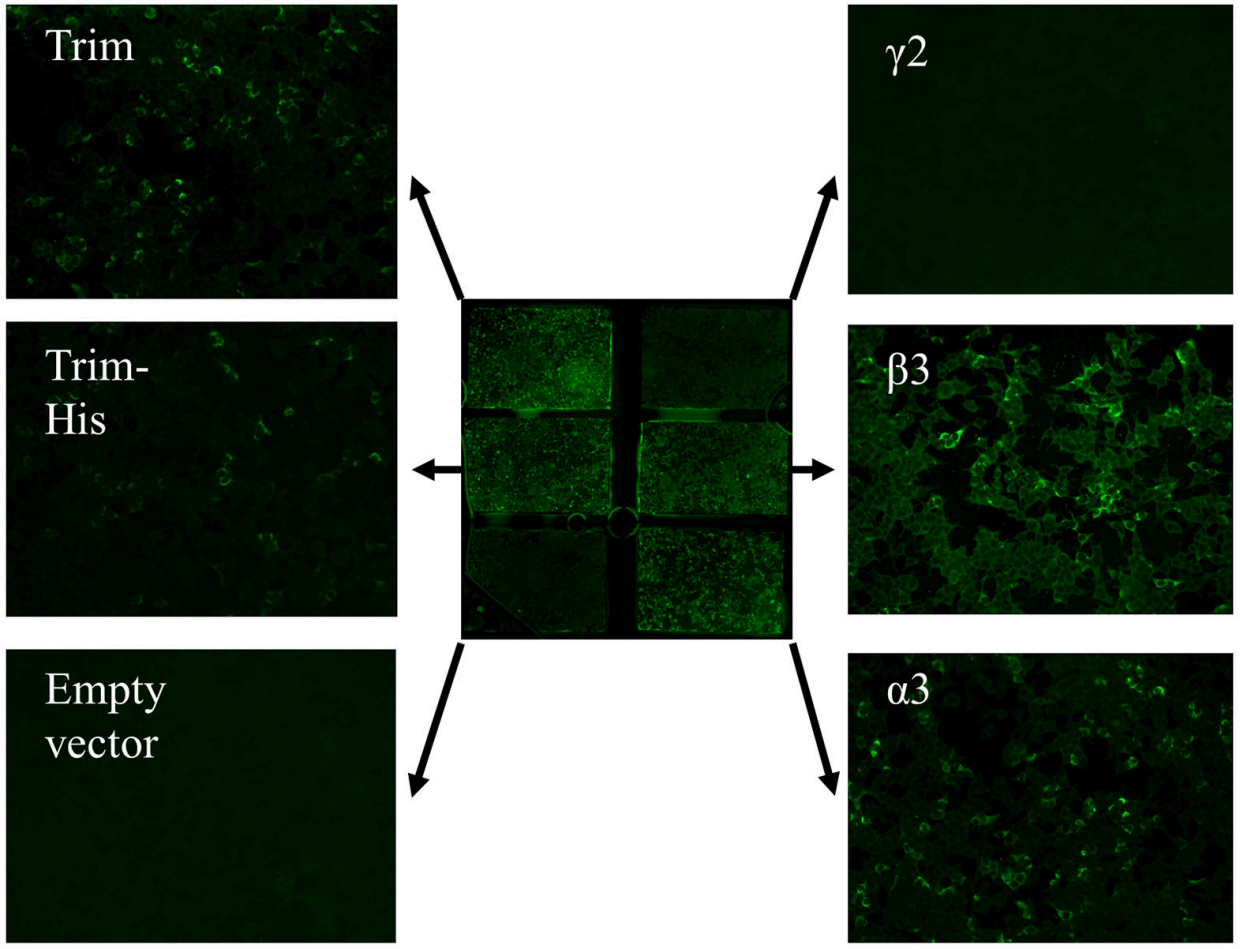
Anti-laminin 332 BIOCHIP® mosaic. The mosaic contains HEK293 cells expressing the recombinant laminin 332 heterotrimer (Trim; upper left and middle left), the recombinant γ2- t β3-, and α3-chains of laminin 332 as well as negative control, cells transfected with the empty vector. Here, antibody binding is seen with the heterotrimer and the β3- and α3-chains in a patient with anti-laminin 332 mucous membrane pemphigoid.

**Figure 6:**
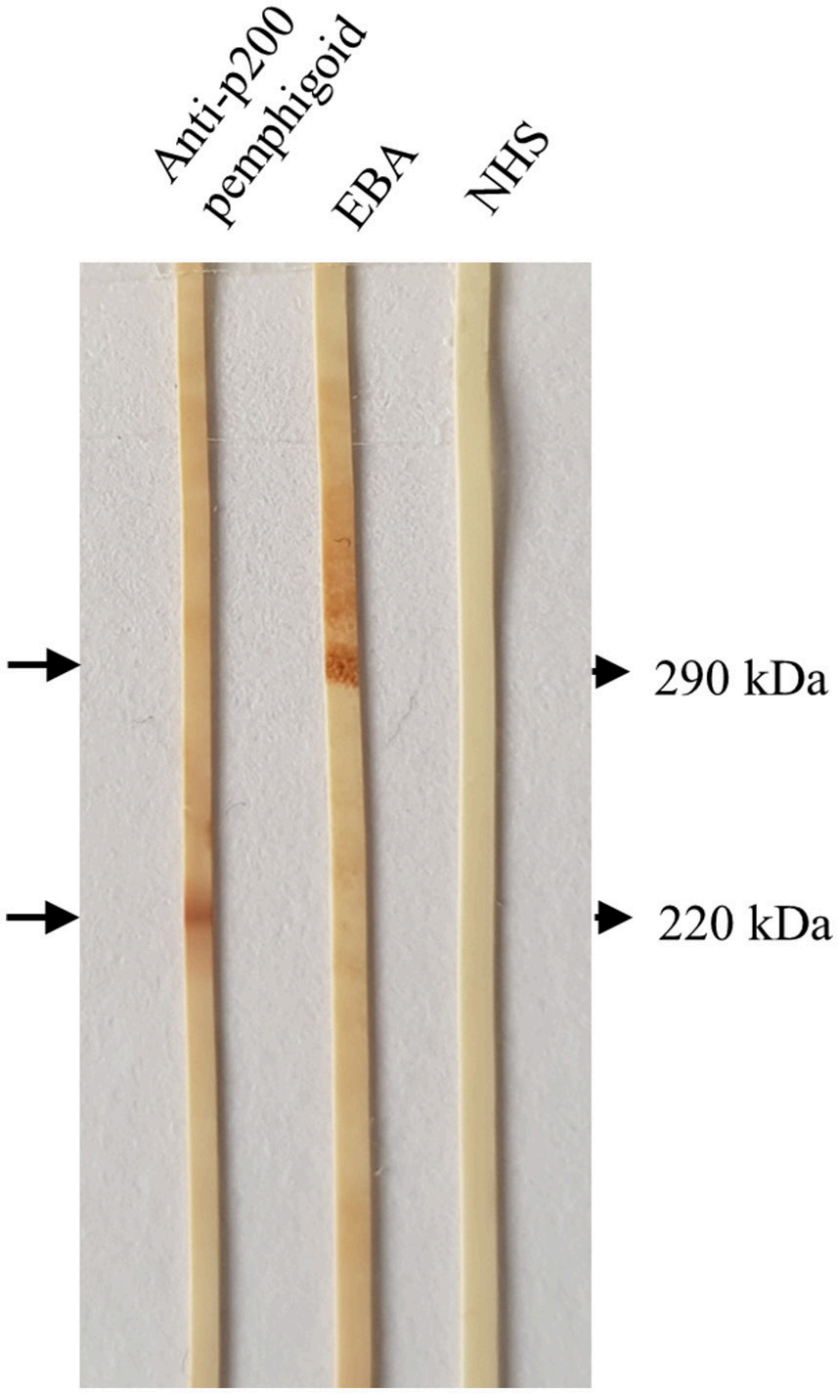
Detection of serum autoantibodies against the p200 protein and type VII collagen by Western blotting with extract of human dermis. Sera from a patient with anti-p200/laminin γ1 pemphigoid and epidermolysis bullosa acquisita (EBA), respectively, showed IgG4 reactivity against the p200 antigen **(lower arrow)** and type VII collagen **(upper arrow)**. In the right lane, a normal human serum (NHS) was applied.

**Figure 7:**
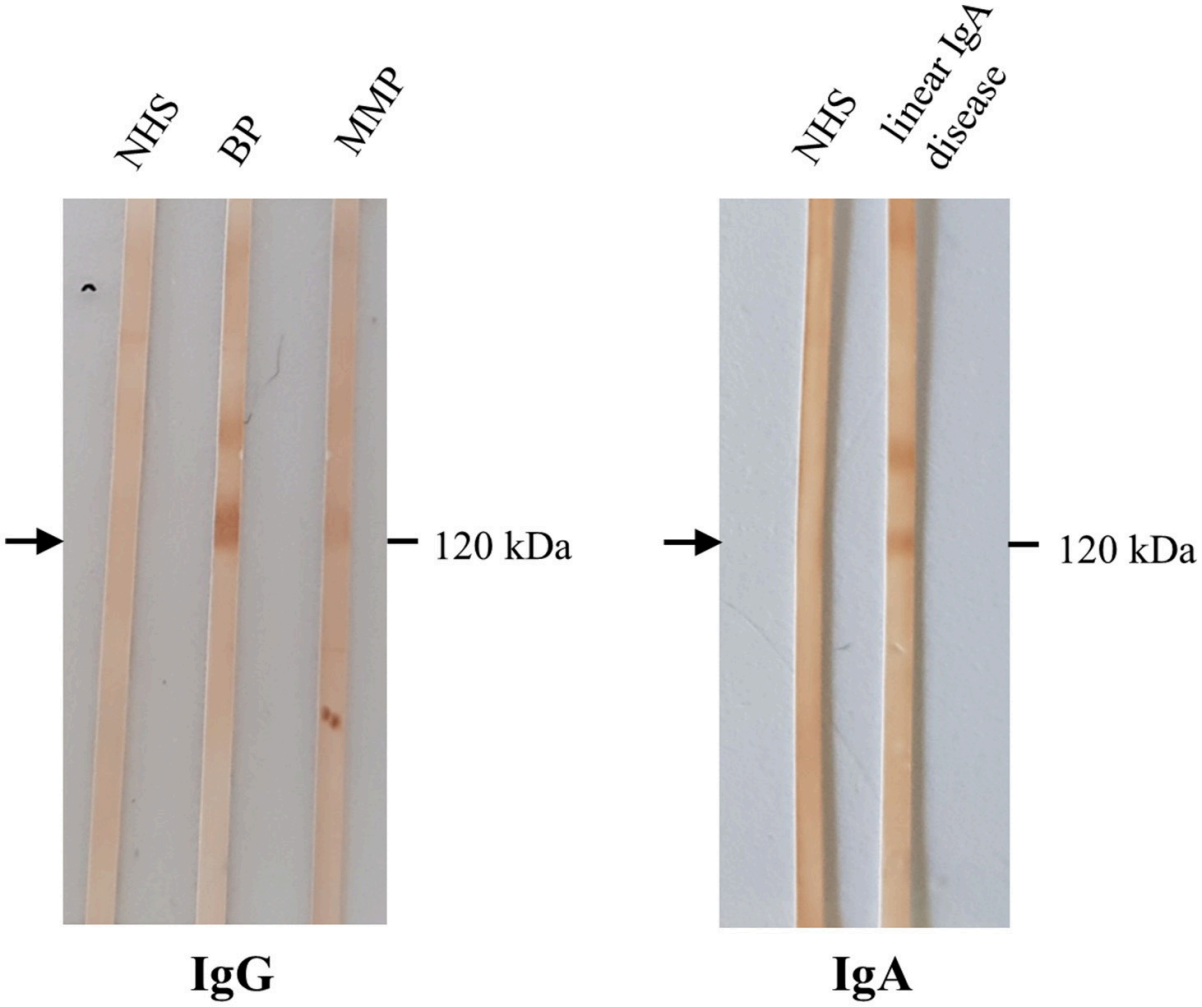
Detection of IgG and IgA autoantibodies against LAD-1 by Western blotting with the concentrated conditioned medium of cultured human keratinocytes. Sera from patients with bullous pemphigoid (BP), mucous membrane pemphigoid (MMP), both without BP180 NC16A IgG by ELISA, showed IgG reactivity with the soluble ectodomain of BP180 (LAD-1; **left**, arrow). In serum of a patient with linear IgA disease, IgA antibodies against LAD-1 were present **(right)**. NHS, normal human serum.

In addition, two multivariant ELISA systems compiled of the individual assays include recombinant Dsg 1 and 3, BP180 NC16A, BP230, type VII collagen, and only in one system, envoplakin, are widely available (75, 76).

(ii) IIF-based assays employing recombinant forms of the target antigens are available as multivariant assays and thus, offer a single-step method for the diagnosis of AIBDs. These assays are based on the BIOCHIP® mosaic technology using normally-sized laboratory slides with 5–10 incubation fields. The serum sample is loaded onto an incubation field, consisting of several miniature biochips coated with different substrates (e.g., monkey esophagus, salt-split skin, recombinant BP180 NC16A or HEK293 cells recombinantly expressing Dsg1, Dsg3, or BP230). We have shown that the sensitivity and specificity of BIOCHIP® mosaic analysis is comparable to that of ELISA systems regarding AIBDs (77). Meanwhile, this technology has been applied in different routine laboratories worldwide (78– 80). More recently, a mosaic comprising 4 biochips coated with recombinant BP180 NC16A, HEK cells expressing BP230, salt-split skin and monkey esophagus, respectively, showed a sensitivity of 100% when testing with pemphigoid gestationis sera (79). A BIOCHIP® mosaic including the immunodominant NC1 domain of type VII collagen yielded sensitivities of 92 and 100% (50, 81), indicating that BIOCHIP® technology is a valuable tool in the routine diagnosis of pemphigoid gestationis and EBA. As for desmocollins, anti-desmocollin IgG and/or IgA reactivity was only found in about 3% of around 400 pemphigus sera, using a BIOCHIP® mosaic containing recombinant forms of desmocollin 1, −2, and −3 (82). Therefore, according to the guidelines of the German Dermatological Society, the analysis for anti-desmocollin reactivity is only recommended in patients with IgA pemphigus, pemphigus vegetans, atypical pemphigus, and the rare patients with pemphigus vulgaris/foliaceus without anti-Dsg reactivity (36). Most recently, a BIOCHIP® mosaic was developed containing recombinant chains of laminin 332 for the diagnosis of anti-laminin 332-pemphigoid (83) (Figure 5). This BIOCHIP® mosaic yields sensitivities between 75 and 85% with a specificity of nearly 100% (83).
(iii) Immunoblotting and immunoprecipitation are performed using recombinant proteins or extracts of dermis, epidermis, bovine gingiva, amnion membrane or cultured keratinocytes (80–86). These systems are part of the diagnostic algorithm for AIBD in some laboratories. They can be used for the detection of anti-p200 autoantibodies (Figure 6), anti-laminin γ1 autoantibodies, antibodies against C-terminal stretches of BP180, and the soluble ectodomain of BP180 (LAD-1; Figure 7), as well as autoantibodies against cell-derived forms of envoplakin, periplakin, desmoplakin, BP180, BP230, α4β6-integrin, laminin 332, and type VII collagen (Figure 6) (87). Latter test systems are, however, only available in specialized laboratories including the autoimmune laboratory of the Department of Dermatology, Lübeck, Germany (88). The laboratory has been accredited by Deutsche Akkreditierungsstelle (DAkkS D-ML-13069-06-00) and is also involved in the development of novel assay systems (www.uksh.de/dermatologie-luebeck/Infos+für+Ärzte+und+Einsender/Autoimmunlabor.html). The main diagnostic algorithm of our laboratory is shown in Figure 4 and further detailed in Schmidt et al. (3).

## Diagnostically relevant clinical and immunopathological characteristics of major AIBD

### Pemphigus vulgaris and pemphigus foliaceus

Pemphigus can be divided in two major clinical subtypes, PV and PF. Autoantibodies in pemphigus are directed against epidermal desmosomes, mainly desmoglein (Dsg) 1 and 3. A common clinical finding is a positive Nikolsky sign. Here, mechanical friction of perilesional skin results in exfoliation of the outermost skin layer. The Nikolsky sign moderately sensitive, but highly specific in the diagnosis of pemphigus (89). PF is clinically characterized by flaccid, superficial erosions preferentially in seborrheic areas. The erosions are usually covered by scaling, which is due to the detachment of the superficial layers of the epidermis (90). In PF, mucous membranes are completely spared (3). Autoantibodies in PF are directed against Dsg1 and can be detected by ELISA or IIF microscopy (46, 88, 91) (Figure 4). In nearly all PF patients, anti-Dsg1 serum levels closely correlate with disease activity (46).

In contrast to PF, patients with PV always suffer from mucous membrane lesions (Figure 2 middle). These are accompanied to a variable extent with blisters and/or erosions of the skin. Autoantibodies in PV are directed against Dsg 3 (92). When in addition to mucosal involvement, lesions are also present on the skin, patients with PV also have autoantibodies against Dsg1 (1). According to the extent of affected skin, three types of PV can be distinguished: (i) the mucosal-dominant type with limited cutaneous involvement (Dsg 3-autoantibodies are predominant), (ii) the mucocutaneous type with both mucosal and cutaneous involvement (Dsg3- and Dsg1-autoantibodies are equally predominant) and the cutaneous type with predominant anti-Dsg1 and pathogenically weak anti-Dsg3 autoantibodies (1). Alike Dsg1-specific autoantibodies, anti-Dsg3-autoantibodies can be detected by ELISA (46). Both Dsg1- and 3-autoantibody levels correlate with the disease activity and can therefore be used as disease activity marker (30, 46).

### Paraneoplastic pemphigus

Paraneoplastic pemphigus (PNP) is an AIBD that is characterized by its association with malignant (or rarely benign) neoplasms. The most frequently associated neoplasms are B-cell lymphoma, Castleman disease, chronic lymphocytic leukemia, thymoma, and Waldenstrom macroglobulinemia (84, 93, 94). The clinical phenotype is diverse. First, PNP mainly affects the oral mucosa with other mucous membranes less frequently involved (95–97). Cutaneous lesions may arise on any part of the skin and may include: (i) pemphigus-like lesions with flaccid blisters, erosions, erythema and crusts; (ii) BP-like lesions such as urticarial lesions and tense blisters (96); (iii) erythema multiforme-like lesions and (iv) lichen planus-like lesions presenting as flat scaly papules and intense mucous membrane involvement (95). Furthermore, pulmonary destruction leading to bronchiolitis obliterans was noticed in many PNP-patients (98).

Apart from Dsg3, the autoantibodies may be directed against plakins such as BP230, periplakin, envoplakin, desmoplakin 1 and 2, and plectin (84). More recently, antibodies against desmocollins, α2 macroglobulin-like 1, and epiplakin have been described (62, 99, 100) (Figure 4). Antibodies against envoplakin and periplakin are most frequent (60, 101, 102). They can be detected via Western blotting or immunoprecipitation of extracts from keratinocytes (84), and, more conveniently, by a commercial ELISA employing the recombinant N-terminus of envoplakin (60).

### Bullous pemphigoid

In BP, autoantibodies are directed against a 180 kDa-sized (BP180/BPAG2/XVII collagen) and/or a 230 kDa-sized (BP230/BPAG1) antigen, which are essential for dermal-epidermal adhesion (103) (Figure 2 left). The disease is mainly diagnosed in people aged between 75 and 80 years (18). It rarely occurs in people under the age of 50 years with few children described with BP (104). In nearly all patients with BP, intense pruritus is present (105). Classically, BP presents with tense blisters and erosions. In contrast to pemphigus, the Nikolsky sign is negative. Alternatively or additionally, urticarial and erythematous non-bullous lesions are present (106). In fact, about 20% of patients present with non-bullous variants with excoriations, erythematous, or urticarial lesions (107). Non-bullous lesions also usually develop during a prodromal stage that may last for several months. Mucosal lesions, which occur in 15–20% of the BP patients, are associated with high disease severity and with absence of anti-BP230 antibodies (108).

BP180 is a collagen-type transmembrane glycoprotein of about 1,500 amino acids. It is a heterotrimer, consisting of a globular intracellular domain, a short transmembranous segment, and an extracellular C-terminal domain composed of 15 collagen repeats that are separated by 16 noncollagenous (NC) subdomains (109). The C-terminal domain forms a loop structure as it goes through the lamina lucida, spans the lamina densa, and then bends back into the lamina lucida (110). The 16th of the extracellular non-collagenous subdomains, NC16A, is the immunodominant region in BP (111). It is used in two ELISA systems (47, 48) (Figure 4), which on the one hand provide a sensitive and specific diagnostic tool for the routine diagnosis of BP and on the other hand are used to monitor the serum levels of anti-BP180 NC16A antibodies during the course of the disease (112). Alternatively, an IIF test using recombinant NC16A is widely available (77) (Figure 4). In most of the BP patients, autoantibodies are also directed against BP180-epitopes outside the NC16A-domain (113). In the 10–15% of BP patients with no reactivity against NC16A, testing for those antibodies is recommended; however, no commercial assay is available so far. In addition to several recombinant fragments of the C-terminal part of BP180, the cell-derived 120 kDa shed ectodomain present in the conditioned concentrated medium of cultures keratinocytes can be applied by immunoblotting for the detection of non-NC16A-reactive sera.

The major immunoglobulin class in BP is IgG. However, it has been shown that some patients also develop anti-BP180 IgA and IgE autoantibodies (114). In fact, most of the BP sera contain both IgG and IgA autoantibodies to BP180 (114, 115). Anti-BP180 IgE antibodies can be detected in 30–95% of the BP patients, and their detection corresponds to a high disease severity (70, 116).

BP230 is an intracellular component of the hemidesmosomal anchoring complex. It is a member of the plakin family. Anti-BP230 IgG can be detected in the serum of 40–60% of the BP patients. Like for BP180, commercial ELISA systems are available for the detection of anti-BP230 antibodies, which can be used for the diagnosis of BP (49, 52) (Figure 4). 80% of the anti-BP230 autoantibodies found in the sera of BP patients most frequently target the globular C-terminal domain of BP230 that interacts with keratin filaments (117, 118). However, it remains unclear whether anti-BP230 antibodies are directly pathogenic (119). Also, unlike anti-BP180, serum levels of anti-BP230-antibodies do not correlate with the disease activity in BP patients (120). However, the detection of anti-BP230 autoantibodies remains a useful tool for the diagnosis of BP. Regarding this matter, the combined use of the BP180 and BP230 ELISA system provides a sensitivity of around 90% for the detection of circulating autoantibodies (52, 56, 57).

### Pemphigoid gestationis

Pemphigoid gestationis (previously called herpes gestationis) is a dermatosis of pregnancy. It usually occurs during the third trimenon and less commonly, in the second trimenon or post partal period (121, 122). In contrast to BP, blisters are infrequent and usually small with predominating urticarial erythema frequently initiating around the umbilicus. Pemphigoid gestationis tends to recur in subsequent pregnancies, appearing earlier and with a more severe course. Serum autoantibodies to BP180 NC16A can be detected in >95% of the patients by ELISA, having a sensitivity of 97% (Figure 4) (47, 55). Recently, Sadik et al. detected NC16A reactivity in all of a large cohort of 65 pemphigoid gestationis sera using an IIF test based on the Biochip® mosaic technology (123). The main IgG subclasses are IgG1 and IgG3, explaining their high potential for the fixation of complement (124), a feature that is exploited by the IIF complement binding test that visualizes complement-binding anti-basement membrane antibodies (Figure 4). Anti-BP230 reactivity is found in only 10% of sera (123).

### Mucous membrane pemphigoid

**Figure 8:**
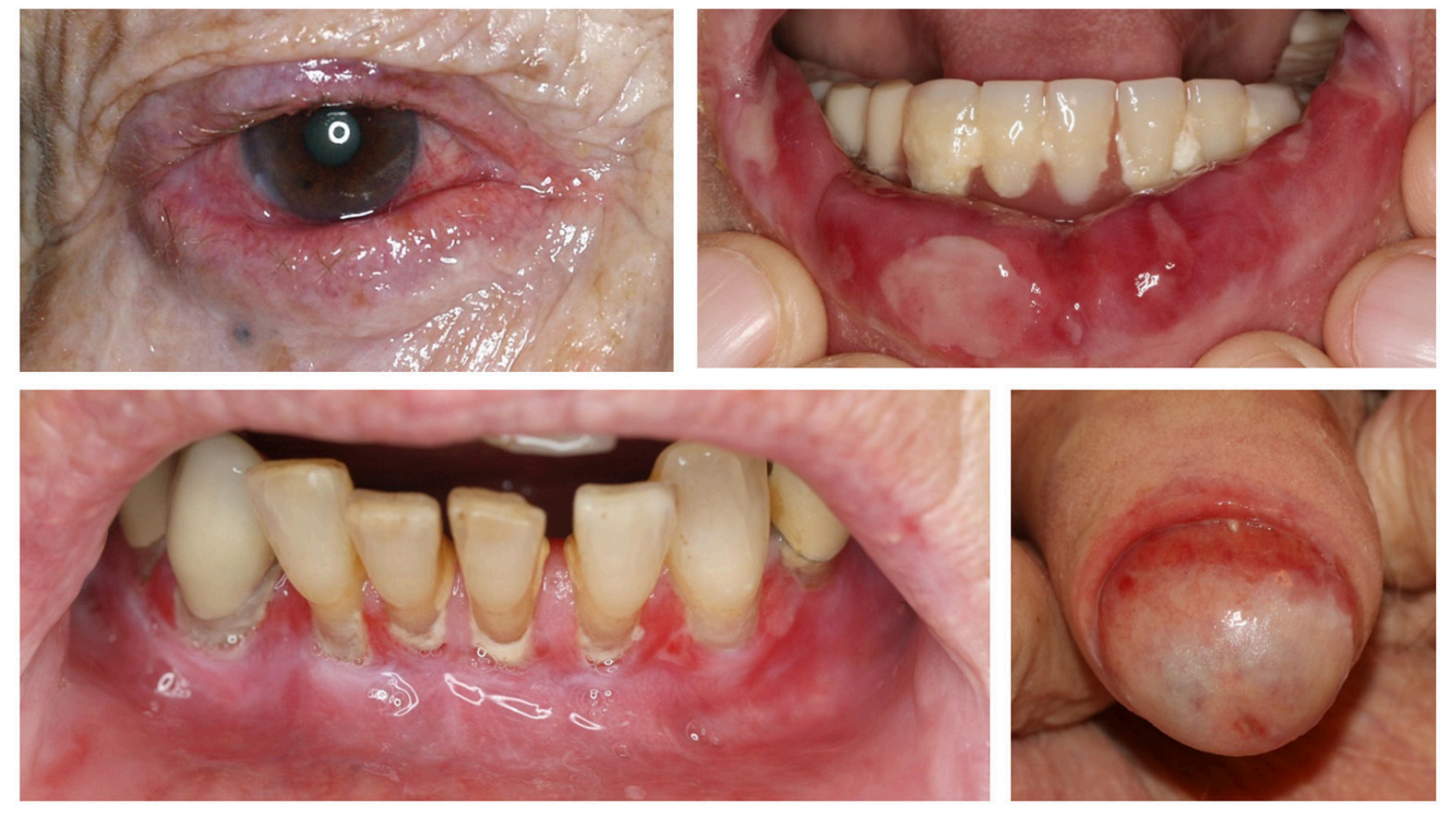
Clinical findings in mucous membrane pemphigoid affecting the conjunctiva, lips, gingiva, and glans penis.

MMP is defined as pemphigoid disease with predominant involvement of mucous membranes (125). It usually affects the mucous membranes of the mouth, eyes and genitals. Complications of the disease are conjunctival involvement and blindness, which may cause serious morbidity (Figure 8) (126, 127). Diagnosis is made by DIF microscopy of a perilesional biopsy, showing linear deposition of IgG and/or IgA and/or C3 along the DEJ (Figure 1) (126). In IIF microscopy, autoantibodies can only be detected in 50% of the MMP patients. Therefore, immunoprecipitation, Western blotting, and ELISA systems that employ cell-derived and recombinant proteins are essential diagnostic tests for MMP (Figure 4) (126). The main target antigen in MMP is BP180. However, in contrast to BP, the NC16A domain is only targeted in around 50%. More commonly, the autoantibodies target C-terminal epitopes of BP180 such as LAD-1, the soluble ectodomain of BP180 (128, 129). Those autoantibodies can be detected by Western blotting, using the respective recombinant fragments of the C-terminus of BP180. Both anti-BP180 IgG and IgA are predominant in anti-BP180-type MMP. Therefore, it is necessary to test for both isotypes (Figure 4) (128). Further antigens in MMP are laminin 332 as well as α6 and β4 integrin. Anti-laminin 332 antibodies can be detected in around 25% of the MMP-patients (128, 130). Laminin 332, which was formerly known as epiligrin or laminin 5, is a heterotrimeric protein composed of an α3, β3, and γ2 subunit. Anti-laminin 332 autoantibodies typically target the α3 chain (131). Since the presence of anti-laminin 332 autoantibodies is associated with development of malignancies in 25% (38, 132, 133), screening for anti-laminin 332 reactivity is strongly recommended in every MMP patient and in case of positivity, a tumor search is mandatory. Unfortunately, no detection system for serum antibodies against laminin 332 is widely available. A sensitive and highly specific assay for serum anti-laminin 332 IgG based on the Biochip® mosaic technology has recently been developed (83) and will be commercialized later in 2018. Antibodies against α6 and β4 integrin were detected in a few cases of oral and conjunctival MMP, respectively (134).

### Linear IgA disease

Linear IgA disease (LAD) is characterized by subepidermal blistering and linear deposition of predominantly IgA at the DEJ. The disease is characterized by its heterogenous phenotype that may be similar to other autoimmune skin blistering diseases. Mostly, the patients present with vesiculobullous lesions on the skin and adjacent mucous membranes (135, 136). Using DIF microscopy of a perilesional biopsy, a linear deposition of IgA autoantibodies along the DEJ can be detected (Figure 1). The autoantibodies bind to antigens of different molecular weights, including 97-, 120-, 180-, 200-, 230-, 280-, 285-, and 290-kDa proteins (137–139). According to the IIF findings, LAD can be divided into the lamina lucida type, where sera react with the epidermal side of salt-split skin and mostly with LAD-1 (140), and the sublamina densa type, that reveals serum antibodies against the dermal side of salt-split skin recognizing type VII collagen (138, 141, 142) (Figure 4). Interestingly, a recent study showed that type VII collagen is also the most common target antigen in vancomycine-induced LAD (143). Due to sematic overlap, patients with IgA reactivity against type VII collagen can also be classified as IgA EBA, a view that is supported by a recent consensus of an international expert panel (144).

### Anti-P200/anti-laminin γ 1 pemphigoid

Anti-p200 pemphigoid is an autoimmune skin blistering disease with antibodies directed against a 200 kDa protein of the DEJ (145). Since laminin γ1 is the target antigen in 90% of the cases, it is also known as anti-laminin γ1 pemphigoid (65). Like LAD, the clinical presentation is heterogeneous and in most of the cases resembles BP and the inflammatory variant of EBA. The lesions heal without scarring or milia formation. Mucous membranes are involved in about 20% of the patients (146). Palmoplantar involvement seems to be more frequent compared to BP and a high association with psoriasis is seen in Japanese patients with anti-laminin 332 pemphigoid (146, 147). Antibodies against p200 bind to the floor of the artificial blister of salt-split skin using IIF microscopy (Figure 4). They can be detected by Western blotting with extracts of human dermis (Figure 6) (145). However, the preparation of those extracts is challenging and problematic. Therefore, an ELISA system was developed, using the recombinant C-terminus of laminin γ1, with a sensitivity of around 70% and a specificity of nearly 99% (64).

### Epidermolysis bullosa acquisita

EBA affects skin and, to less extent, mucous membranes and is characterized by autoantibodies against type VII collagen (Figure 2 right) (31, 148). There are two main clinical forms of EBA (144, 149, 150). The mechanobullous form represents the classical form of EBA. It is clinically characterized by skin fragility, tense blisters, vesicles, and erosions on non-inflamed skin in trauma-prone sites. Lesions may heal with scarring and milia formation (149, 151). In about two thirds of EBA, the inflammatory variant develops resembling BP, MMP, or LAD (149, 152). The diagnostic gold standards are direct immunogold electron microscopy, a methods nowadays only performed for this purpose in handful of centers, and more conveniently, DIF microscopy (144). By latter method, diagnosis of EBA can be made when a u-serrated binding pattern is present as detailed above (Figures 1, 3). Type VII collagen-specific autoantibodies are deposited at the floor of the artificial blister of salt-split human skin using IIF microscopy (Figure 2 right) and are mostly directed against the noncollagenous (NC)1 domain (153, 154). Autoantibodies can be detected via Western blotting, using an extract of the human dermis (Figure 6). Three assays for the diagnostic detection of serum IgG against type VII collagen are available; two ELISA system using the NC1 domain or both the NC1 and NC2 domains as well as an IIF test based on the Biochip mosaic technology employing human cells that express the recombinant NC1 domain (Figure 4) (50, 51, 75). Anti-type VII collagen ELISA values were shown to correlate with disease activity (155), thus, like in PV, PF, and BP, the respective ELISA systems are useful tools not only for the diagnosis of the disease but also to guide treatment decisions during the course of the diseases. Patients with predominant or exclusive IgA reactivity against type VII collagen are usually classified as IgA EBA following the consensus of an international expert panel (144, 149, 152, 156, 157).

### Dermatitis herpetiformis

Dermatitis herpetiformis is an autoimmune disease that always occurs in combination with glutensensitive enteropathy (celiac disease). It is clinically characterized by grouped vesicles and papules and predominantly affects the elbows, buttocks, and knees (146, 147, 149, 158–160). The autoantigen is the epidermal transglutaminase, however antibodies against gliadin, endomysium, tissue transglutaminase (TG2), and epidermal transglutaminase (TG3) can be detected (161). The main antibody-subclass is IgA but can be IgG in some patients. In patients under treatment with dapsone or on gluten-restricted diet, autoantibodies against the epidermal transglutaminase are found more frequently than autoantibodies to tissue transglutaminase (162). The diagnosis is based on the DIF and IIF microscopy findings as well as commercial ELISA systems. Here, IgA (or IgG)-autoantibodies against the epidermal and tissue transglutaminase as well as the deaminated gliadin-analogous fusion (GAF) peptides can be detected (61, 162).

## Author contributions

MW and ES performed the literature research, acquired and designed the figures and wrote the manuscript. DZ performed literature research and critically revised the manuscript.

### Conflict of interest statement

DZ and ES have a scientific cooperation with Euroimmun, Lübeck. The remaining author declares that the research was conducted in the absence of any commercial or financial relationships that could be construed as a potential conflict of interest.
